# The Complete Mitochondrial Genomes of Three Sphenomorphinae Species (Squamata: Scincidae) and the Selective Pressure Analysis on Mitochondrial Genomes of Limbless *Isopachys gyldenstolpei*

**DOI:** 10.3390/ani12162015

**Published:** 2022-08-09

**Authors:** Lian Wu, Yao Tong, Sam Pedro Galilee Ayivi, Kenneth B. Storey, Jia-Yong Zhang, Dan-Na Yu

**Affiliations:** 1 College of Chemistry and Life Science, Zhejiang Normal University, Jinhua 321004, China or; 2 Department of Biology, Carleton University, Ottawa, ON K1S5B6, Canada; 3 Key Lab of Wildlife Biotechnology, Conservation and Utilization of Zhejiang Province, Zhejiang Normal University, Jinhua 321004, China

**Keywords:** positive selection, mitochondrial genomes, *Isopachys gyldenstolpei*, limbless skinks

## Abstract

**Simple Summary:**

Skinks are the most species-rich group of lizards and are widely distributed around the world. The family Scincidae (Reptilia: Lacertiformes) includes limbed and limbless representatives occupying diverse habitats and showing a range of morphologies. Both limbed and limbless skinks have unique locomotion patterns in their habitats. Locomotion is the process of energy consumption, of which different modes may have different energy demands. As the center of energy metabolism in organisms, mitochondria provide most of the energy for physiological and biochemical activities via oxidative phosphorylation. Here, we employed mitochondrial genomes to investigate potential selective pressures among limbless skinks. *Isopachys gyldenstolpei*, as a typical limbless skink, has a different locomotion pattern compared to a limbed skink. Thus, *I. gyldenstolpei* can be used to study whether limb loss has a positive selection on mitochondrial genes. Two typical limbed skinks, *Sphenomorphus indicus* and *Tropidophorus hainanus*, were included in this study to compare the selective pressure analysis on mitochondrial genomes. In addition, the phylogenetic relationships within Scincidae are also discussed.

**Abstract:**

In order to adapt to diverse habitats, organisms often evolve corresponding adaptive mechanisms to cope with their survival needs. The species-rich family of Scincidae contains both limbed and limbless species, which differ fundamentally in their locomotor demands, such as relying on the movement of limbs or only body swing to move. Locomotion requires energy, and different types of locomotion have their own energy requirements. Mitochondria are the energy factories of living things, which provide a lot of energy for various physiological activities of organisms. Therefore, mitochondrial genomes could be tools to explore whether the limb loss of skinks are selected by adaptive evolution. *Isopachys gyldenstolpei* is a typical limbless skink. Here, we report the complete mitochondrial genomes of *I*. *gyldenstolpei*, *Sphenomorphus indicus,* and *Tropidophorus hainanus*. The latter two species were included as limbed comparator species to the limbless *I. gyldenstolpei*. The results showed that the full lengths of the mitochondrial genomes of *I*. *gyldenstolpei*, *S*. *indicus*, and *T. hainanus* were 17,210, 16,944, and 17,001 bp, respectively. Three mitochondrial genomes have typical circular double-stranded structures similar to other reptiles, including 13 protein-coding genes, 22 transfer RNAs, 2 ribosomal RNAs, and the control region. Three mitochondrial genomes obtained in this study were combined with fifteen mitochondrially complete genomes of Scincidae in the NCBI database; the phylogenetic relationship between limbless *I*. *gyldenstolpei* and limbed skinks (*S*. *indicus* and *T*. *hainanus*) is discussed. Through BI and ML trees, Sphenomorphinae and Mabuyinae were monophyletic, while the paraphyly of Scincinae was also recovered. The limbless skink *I*. *gyldenstolpei* is closer to the species of *Tropidophorus,* which has formed a sister group with (*T*. *hainanus + T*. *hangman)*. In the mitochondrial genome adaptations between limbless *I. gyldenstolpei* and limbed skinks, one positively selected site was found in the branch-site model analysis, which was located in ND2 (at position 28, BEB value = 0.907). Through analyzing the protein structure and function of the selected site, we found it was distributed in mitochondrial protein complex I. Positive selection of some mitochondrial genes in limbless skinks may be related to the requirement of energy to fit in their locomotion. Further research is still needed to confirm this conclusion though.

## 1. Introduction

Scincidae, as the most species-rich family in Squamata, has 1744 species currently recorded on the Reptile Database (http://www.reptile-database.org/, accessed on 22 March 2022) [1]. All live in tropical and temperate regions and can be found on all continents, except for Antarctica and most oceanic islands [2]. With such a wide distribution, it is not surprising that skinks exhibit diverse morphological characteristics to adapt to their various distribution environments [3,4]. Skinks range from fully pentadactyl forms to completely limbless body forms found in Scincidae [1]. Compared with the wide habitat range of limbed skinks, most limbless skinks are cryptozoic, mainly choosing to inhabit underground burrows or under rocks, with little exposure above ground [5,6]. At the same time, different movement abilities between limbed and limbless skinks have arisen in adapting to their habitats. Limbed body forms are often associated with high dispersal ability in Scincidae, whereas limbless body forms show reduced patterns of dispersal. The secretive living environments of the skink species and the scarcity of available samples have led to a poor understanding of the diversity, geographical distribution, genetic relationships, and phylogeny of these groups [7].

*Isopachys gyldenstolpei* belongs to the subfamily Sphenomorphinae and is mainly distributed in Thailand and Burma [8]. As an elongated, fossorial, and limbless lizard, *I. gyldenstolpei* burrows in dry soil covered with dead leaves and sticks [8,9]. Due to a scarcity of samples, no complete mitochondrial genome of *I. gyldenstolpei* has been reported to date. Compared with limbless skinks, the limbed skinks are not only more widely distributed, but also have diverse lifestyles. Thus, there is much interest in the typical morphological characteristics of limb loss and axial elongation in Scincidae. For example, the species-rich genus, *Sphenomorphus*, shows diverse lifestyles in a wide array of habitats, ranging from upland cloud forests to small, arid, virtually barren islands, to lowland and hill forests dominated by Dipterocarpus [10,11,12]. In contrast, lizards of the genus *Tropidophorus* are semi-aquatic and mainly dwell in lowlands near mainland forest streams [13,14,15]. The present study sequenced the mitochondrial genome of one limbless skink, *I. gyldenstolpei*, followed by the mitochondrial genomes of two limbed skinks *Sphenomorphus indicus* and *Tropidophorus hainanus*. Differences in the mitochondrial genome were compared to the morphological disparity between limbed and limbless skinks. At present, the monophyly of Scincidae has been widely recognized, and the taxonomic controversy is mainly focused on the monophyly of the subfamily Scincinae [16,17,18,19]. As an ideal molecular marker, mitochondrial genes have been widely used to solve the phylogeny of Scincidae [20]. Mitochondrial genomes were used to discuss the phylogenetic status of limbless *I. gyldenstolpei* and mitogenomes could similarly provide useful clues for the remaining disputes in the phylogenetic relationships of Scincinae.

Moving is an energy-expensive process, and different models of movement require different levels of energy consumption. Losing legs or damage to appendages associated with movement may impose additional energetic costs [21]. As the energy-producing center of organisms, up to 95% of the ATP used by eukaryotic cells is provided by mitochondria through oxidative phosphorylation (OXPHOS) [22,23,24,25]. The mitochondrial genomes of lizards have the same double-circular structures as those of other vertebrates, with typical lengths of 16–19 kb and conserved genetic compositions, including 13 protein-coding genes, 22 tRNAs, 2 rRNAs, and a major non-coding control region [22,26]. Due to its function in energy metabolism, mitochondrial DNA has attracted much attention for its selective role in adaptive evolution, and some studies have shown that the differences in locomotive ability are reflected in the different selection mechanisms of mitochondrial genomes [24,25,27,28,29,30]. For example, compared with flightless grasshoppers, flying grasshoppers have been positively selected for their mitochondrial genes in response to the energy requirements of flight [31]. Among fish, it has been found that the amount of swimming and energy consumption are different between migratory fishes and nonmigratory fishes, and genes related to the energy regulation in mitochondria have shown evidence for a direct response to selective pressure [24]. In birds, the mitochondrial genes of fast-flying birds have different effective energy metabolic requirements than those of weak-flying or flightless species, suggesting that evolution constrains the mitochondrial DNA of birds with different motor abilities, as has also been found in mammals [25]. Similarly, the mitochondrial genes of plateau birds were positively selected after adaptation to the plateau environment [32]. Research on the origins of flight in bats has shown that mitochondrial genes involved in energy metabolism were naturally selected to adapt to the huge changes in energy requirements during the origins of flight (as compared to other mammals) [28]. Selection pressures on mitochondrial genes have also been found in limb-reduced *Dibamus bourreti* and other limbless squamates, suggesting that selection pressures on mitochondrial genomes may play important roles in the energetic differences in locomotion between limbed and limbless squamates [33]. Therefore, considering that *I. gyldenstolpei* has morphological characteristics related to a complete loss of limbs, we speculate that the mitochondrial genes of limbless skinks may undergo positive selections compared to limbed skinks.

## 2. Material and Methods

### 2.1. Specimen Collection

The soak specimen of *I. gyldenstolpei* was housed in the Animal Herbarium of Zhejiang Normal University, which was collected from Myanmar in 2000. Specimens of *S*. *indicus* and *T*. *hainanus* were collected in Cixi, Zhejiang and Jinxiu, Guangxi, China, respectively. Our experimental procedures complied with current regulations on animal welfare and research in China. The Animal Research Ethics Committee of Zhejiang Normal University approved the experimental design (ZSDW2021057).

### 2.2. DNA Extraction, PCR Amplification, and Sequencing

Total genomic DNA of the three skinks was extracted from tail muscles using an Ezup Column Animal Genomic DNA Purification Kit (Sangon Biotech Company, Shanghai, China). Common primers [34] for lizards were used to amplify several partial segments, and species-specific primers were designed to complete the remaining gaps using Primer Premier 5.0 [35] based on previously obtained sequences (Table S1). The methods described in Zhang et al. [36] were used to amplify both PCR (product length < 3000 bp) and long-range-PCR (product length > 3000 bp). All PCR products were sequenced using the bi-directional primer-walking method by Sangon Biotech Company (Shanghai, China).

### 2.3. Mitochondrial Genome Annotation and Sequence Analyses

Sequences were checked and assembled using DNASTAR Package V.7.1 [37]. The tRNA genes of the sequences were annotated using the MITOS web server (http://mitos.bioinf.uni-leipzig.de/index.py, accessed on 15 December 2021) [38], and their secondary structures were predicted by the tRNAscan-SE online search server [39]. We identified the two rRNA genes (12S and 16S rRNA) and 13 protein-coding genes (PCGs) by comparing them with the complete mitochondrial genomes of other Scincidae in the Clustal W program of Mega 7.0 [40]. The CG View online server V 1.0 [41] was used to draw the maps of complete mitochondrial genomes (https://cgview.ca/, accessed on 15 January 2022). According to the formula: AT skew = (A − T) ÷ (A + T), GC skew = (G − C) ÷ (G + C), we calculated the CG and AT skews in PhyloSuite 1.2.2 [42]. Similarly, PhyloSuite 1.2.2 was also used to calculate the AT content, codon usage, and relative synonymous codon usage (RSCU) of protein-coding genes, and graphically drawn by Adobe Illustrator CS4 [43].

### 2.4. Phylogenetic Analyses

To explore the phylogenetic relationship of Scincidae, we prepared a dataset comprised of the complete mitochondrial genomes of the three skink species in this study and 15 skink mitochondrial genomes available in NCBI, including from Sphenomorphinae (9), Scincinae (5), and Mabuyinae (1) of Scincidae [44,45,46,47,48,49,50,51,52,53,54,55,56], as well as the two outgroups species belonging to Xantusiidae (1) and Cordyloidea (1) [57,58] (Table 1).

**Table 1:** Information about the samples used in this study and the NCBI GenBank accession numbers.

Family	Subfamily	Species	Length	Accession No.	Reference
Scincidae	Mabuyinae	*Eutropis multifasciata*	17,062 bp	MN938934	[44]
	Scincinae	*Ateuchosaurus chinensis*	16,840 bp	MW327509	[45]
		*Plestiodon chinensis*	17,175 bp	KT279358	[46]
		*Plestiodon elegans*	17,304 bp	KJ643142	[47]
		*Plestiodon liui*	17,643 bp	MT662111	[48]
		*Plestiodon tunganus*	17,263 bp	MK370739	[49]
	Sphenomorphinae	*Ablepharus himalayanus*	17,304 bp	MN885892	[59]
		*Isopachys gyldenstolpei*	17,210 bp	MH020638	This study
		*Scincella huanrenensis*	17,212 bp	KU507306	[50]
		*Scincella modesta*	17,466 bp	MN702771	[51]
		*Scincella modesta*	17,511 bp	MN786972	[52]
		*Scincella reevesii*	15,424 bp	MN832615	[53]
		*Scincella vandenburghi*	17,103 bp	KU646826	[50]
		*Sphenomorphus indicus*	16,944 bp	OM117611	This study
		*Sphenomorphus indicus*	17,027 bp	MK450438	[55]
		*Sphenomorphus incognitus*	17,417 bp	MH329292	[54]
		*Tropidophorus hainanus*	17,001 bp	OM117612	This study
		*Tropidophorus hangnam*	16,777 bp	MN977920	[56]
Cordyloidea	Cordylidae	*Smaug warreni*	17,184 bp	NC005962	[57]
Xantusiidae	Xantusiinae	*Lepidophyma flavimaculatum*	16,158 bp	NC008775	[58]

Based on a dataset of the nucleotide sequences of 13 (PCGs), we constructed the Bayesian inference (BI) and maximum likelihood (ML) phylogenetic trees of Scincidae. MAFFT V. 7.475 [60] was used for the alignment of the nucleotide sequences of the 13 PCGs. Then the conservative region was detected by Gblock 0.91b [61] using the default setting. Because the third codon was saturated, our phylogenetic trees were constructed with the 1st and 2nd codons; the 1st and 2nd data were extracted and reserved using MEGA7.0 [40]. Based on the Bayesian information criterion (BIC), the program PartitionFinder 2.2.1 [62] was used to determine the best partitioning scheme and substitution model (Table S2). Considering that RAxML allows for only a single model of rate heterogeneity in partitioned analyses, the ML analysis was performed in RAxML 8.2.0 [63] using the GTR + I + G model, with branches of each node supporting the evaluation under 1000 ultrafast replications. The BI analysis was performed in MrBayes version 3.2 [64] using the partitioning results (Table S2) and was set for 10 million generations with sampling every 1000 generations. The first 25% of generations were discarded as burn-ins. When the phylogenetic tree converged (i.e., when the value of the average standard deviation of the split frequency was stable and the balance was less than 0.01), the tree was harvested.

### 2.5. Detecting Selective Pressure

The selective pressure analysis of genes was mainly obtained by calculating the replacement ratio of nonsynonymous codons and synonymous codons in the protein-coding sequence, namely, *ω* = *dN/dS*, where *ω* = 1 represents neutral mutation, *ω* < 1 means negative selection, and *ω* > 1 indicates positive selection [65]. As an interactive visualization tool, EasyCodeML can be used to detect the selection pressure of 13 PCGs in the Scincidae mitochondrial genomes [66]. In order to investigate whether the mitochondrial genes of *I. gyldenstolpei* were under positive selection, this limbless skink was chosen as the foreground branch and other limbed skinks as the background branch. Selection pressure analysis was conducted using 11,373 nucleotide sites (excluding the initiation and termination codons) and 3791 amino acid sites after alignment of the 13 PCGs from 18 species of Scincidae. Four analytical methods were conducted to explore the mitochondrial gene adaptative evolution of limbless skinks. The branch model mainly defined the heterogeneity of *ω* values of different lineages in the phylogenetic tree, and we observed whether the *ω* values of foreground branches and background branches were significantly different by comparing the one-ratio model and two-ratio model. The site model was used to compare the selection pressure of amino acid sites. The *ω* values of the branch-site model were different between the selected sites, and it was also assumed that the *ω* values of the branches were different, which was mainly used to detect the influence of positive selection on some sites in the foreground branch (model A vs. model A null). The clade model was mainly used to analyze multiple clades, simultaneously [65]. The posterior probability of these models and selected loci were evaluated using the likelihood ratio test (LRTs) and Bayesian empirical Bayes (BEB), respectively. The structural and functional information of selected genes were obtained in UniProt [67], and the three-dimensional structures of the corresponding proteins were constructed by the SWISS-MODEL workspace [68].

## 3. Results

### 3.1. Organization and Characteristics of Three Skink Mitochondrial Genomes

**Figure 1:**
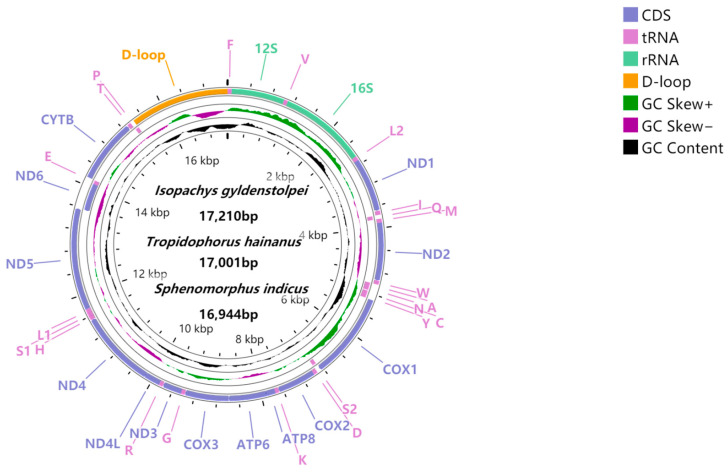
Circular visualization maps of the complete mitochondrial genomes of *I*. *gyldenstolpei*, *T*. *hainanus*, and *S*. *indicus* (since the mitochondrial genomes of the three skinks are similar in structure, a map was used to represent the three skinks). The three circles from the outside to the inside show the gene map (PCGs, rRNAs, tRNAs, and the AT-rich region), the GC content, and GC skew, respectively. Among them, the genes outside the map are coded on the majority strand (J-strand), whereas the genes inside the map are coded on the minority strand (N-strand).

The lengths of the complete mitochondrial genomes were found to be *I. gyldenstolpei* 17,210 bp, *S*. *indicus* 16,944 bp, and *T. hainanus* 17,001 bp, and their corresponding GenBank accession numbers are MH020638, OM117611, and OM117612, respectively. With the typical circular structure, the mitochondrial genome arrangements of the three skink species are the same as those of other reptiles and include 13 PCGs (ND1-6, ND4L, COI-III, Cyt *b*, ATP6, and ATP8), 22 tRNAs, and two rRNA genes (12S rRNA and 16S rRNA), along with a non-coding region (D-loop) between tRNA-Pro and tRNA-Phe (Figure 1). Among the 37 genes, ND6 and 8 tRNAs (including tRNA-Gln, tRNA-Ala, tRNA-Asn, tRNA-Cys, tRNA-Tyr, tRNA-Ser, tRNA-Glu, and tRNA-Pro) are coded on the minor strand (N strand) and the remaining 28 genes are coded on the major strand (J strand). A + T bias of the whole mitochondrial genome was found in all three skinks, being 56% in *I*. *gyldenstolpei*, 57.9% in *S*. *indicus*, and 54.2% in *T*. *hainanus*. Positive AT skews and negative GC skews were found in all three mitochondrial genomes (Table 2).

Gene spacers and overlaps were found between adjacent genes in all three mitochondrial genomes. Nine gene-spacing regions were found in *I*. *gyldenstolpei* and *S*. *indicus*, and eight were found in *T*. *hainanus*, all with lengths from 1 to 14 bp. The longest length of the gene-spacing regions (14 bp) was between tRNA-Asn and tRNA-Cys in all three mitochondrial genomes. Eight, nine, and eleven base pair overlaps were found in the mitochondrial genomes of *I*. *gyldenstolpei*, *S*. *indicus*, and *T*. *hainanus*, respectively, with the lengths ranging from 1 to 10 bp. The longest overlap (10 bp) was between ATP6 and ATP8 in all three skinks.

Among the three complete mitochondrial genomes, the total lengths of the 13 PCGs were 11,382 bp in *I*. *gyldenstolpei* and *S*. *indicus*, and 11,379 bp in *T*. *hainanus*. After comparing the three sequences, two start codons (ATG and GTG) were used in the 13 PCGs. As the most common start codon, ATG was used in most protein-coding genes (11 genes in *I*. *gyldenstolpei* and *T*. *hainanus*, 12 genes in *S*. *indicus*), whereas GTG was used only in COI (*I*. *gyldenstolpei*, *S*. *indicus*, and *T*. *hainanus*), ND1 (*T*. *hainanus*), and ND3 (*I*. *gyldenstolpei*). TAN (TAA/TAG) was often found in the three sequences as the stop codon (eight protein-coding genes). In addition, AGG/AGA was used as the stop codon in some protein-coding genes: COI and ND6 in all three mitochondrial genomes, and COII only in *I*. *gyldenstolpei*. An incomplete stop codon T was found in three protein-coding genes: COIII and ND4 in all three mitochondrial genomes, and COII of *S*. *indicus* and *T*. *hainanus* (Tables S3–S5).

**Figure 2:**
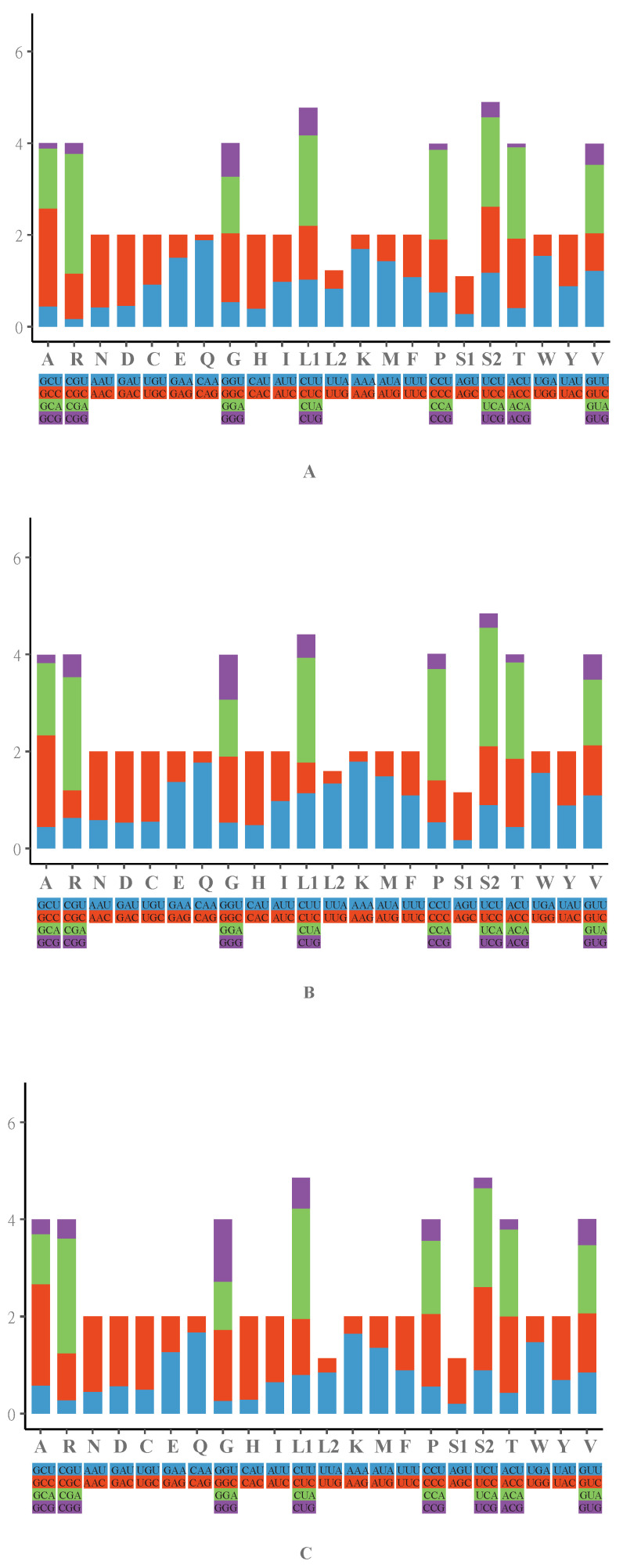
The relative synonymous codon usage (RSCU) of the mitochondrial genomes of *I*. *gyldenstolpei* (**A**), *S*. *indicus* (**B**), and *T*. *hainanus* (**C**). Acronyms stand for different amino acids. The *x*-axis represents all codons used and different combinations of synonymous codons, and the RSCU values are listed on the *y*-axis.

Figure 2 and Supplementary Table S6 show the relative synonymous codon usage (RSCU) and amino acid composition of the three mitochondrial genomes. Throughout the data, A and C nucleotides were the most abundant in the base composition of the three skinks and especially appeared more frequently at the third codon position of all three skinks. The utilization rate of the CUA (Leu1) codon was the highest in the three skinks, which was used 213 times in *I*. *gyldenstolpei*, 231 times in *S*. *indicus*, and 249 times in *T*. *hainanus*. In addition, the use ratio of AUC (Ile), AUA (Met), ACC (Thr), ACA (Thr), and GCC (Ala) was relatively high in all three skinks. At the same time, the utilization rates of some amino acids for the synonymous codon in these three skinks were different, such as UCA (Ser2) in *S*. *indicus* 2.45 > *T*. *hainanus* 2.04 > *I*. *gyldenstolpei* 1.95. Furthermore, A + T bias also existed in 13 PCGs of the three mitochondrial genomes (Table 2).

The two ribosomal RNAs (12S rRNA and 16S rRNA) were separated by tRNA-Val and were found on the H-strand. The full sizes of 12S rRNA were 949 bp (*I*. *gyldenstolpei*), 942 bp (*S*. *indicus*), and 958 bp (*T*. *hainanus*), respectively. Similarly, the total lengths of 16S rRNA for the three skinks were 1558 bp (*I*. *gyldenstolpei*), 1528 bp (*S*. *indicus*), and 1527 bp (*T*. *hainanus*). The sizes of all tRNA genes in the three skinks ranged from 64 to 76 bp. Compared with other tRNAs of the three sequences that showed typical cloverleaf structures, tRNA-Ser1 missed the dihydrouridine (DHU) arm (Figures S1–S3). The total lengths of tRNA in *I*. *gyldenstolpei*, *S*. *indicus*, and *T*. *hainanus* were between 1523 and 1542 bp.

With lengths of 1774 bp *(I. gyldenstolpei*), 1566 bp (*S. indicus*), and 1615 bp (*T. hainanus*), respectively, all control regions of the three skinks could be found between tRNA-Pro and tRNA-Phe. The A + T content of all three skink mitogenomes was more abundant, all greater than 60%. Positive AT skews and negative CG skews were also found in the control regions of the three skinks (Table 2).

### 3.2. Phylogenetic Relationships

**Figure 3:**
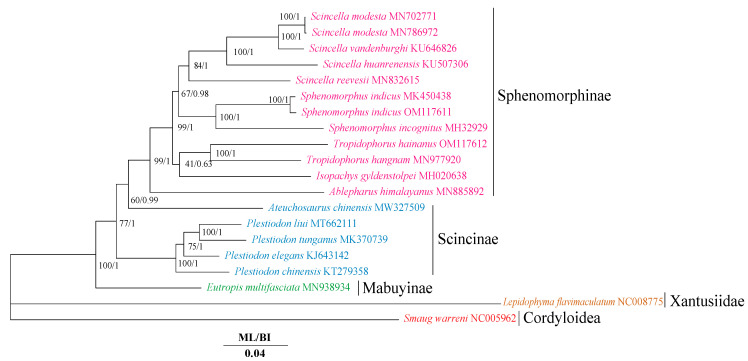
Phylogenetic relationships among 18 species of Scincidae based on the nucleotide dataset of the 13 mitochondrial protein-coding genes. *Smaug warreni* and *Lepidophyma flavimaculatum* were used as the outgroups. The numbers above the branches specify bootstrap percentages from ML (left) and posterior probabilities as determined from BI (right). The GenBank accession numbers of all species are shown in the figure. Different colors represent different subfamilies.

Both BI and ML analyses recovered a consistent topology (Figure 3). In general, except for the outgroups, skinks in this study clustered mainly in three subfamilies: Sphenomorphinae, Scincinae, and Mabuyinae. Sphenomorphinae and Mabuyinae were recovered as monophyletic groups in both BI and ML trees. A sister-group relationship between Mabuyinae and the clade of (Sphenomorphinae + Scincinae) was supported in the present study. In ML and BI trees, the subfamily Scincinae is paraphyletic because *Ateuchosaurus* of Scincinae is nested into Sphenomorphinae. In the subfamily Sphenomorphinae, the limbless *I*. *gyldenstolpei* was a sister clade of (*T. hainanus + T. hangnam*) and then clustered in a clade with (*Sp. indicus + Sp. incognitus*) *+* (*Sc. reevesii +* (*Sc. huanrenensis +* (*Sc. vandenburghi + Sc. Modesta*)))). In ML and BI trees, *Ab. himalayanus* is considered the sister group to all other Sphenomorphinae species.

### 3.3. Positive Selection Analysis of 13 Protein-Coding Genes

The BI tree was used to analyze the selection pressure of 13 protein-coding genes. The results showed that *LRT p*-values were 0.859 and 0.704 in the branch and clade models, respectively. Thus, there were no significant selected sites found in the two models.

The analysis results of the branch-site model and the site model are shown in Table S7. Model A vs. model A null was significant (*p* < 0.05) in the branch-site model, where there was an amino acid selected site and the BEB value was 0.907 (amino acid residue 2017). Amino acid residue 2017 corresponds to amino acid position 28 of ND2 (Figure S4 and Table S8). At position 2017, amino acids were mutated from Leu (L) of the background branch to Thr (T) of the foreground branch. The positive selection site was found in mitochondrial complex I. Position 28 of the ND2 gene was found to be located in the domain of the proton-conducting membrane transporter through protein functional analysis. Two amino acid sites were positively selected (BEB > 0.90, positions 2416 and 3742) in the site model, with the LRT of M7-M8 showing high significance (*p <* 0.01). Amino acid residue 2416 corresponded to amino acid position 83 of the ND3 gene, whereas position 3742 corresponded to amino acid position 124 of the ND6 gene (Table S8). Positive selection sites of mitochondrial genes were found in the site model, which may suggest that some amino acid sites in some skinks have undergone adaptive selection.

## 4. Discussion

### 4.1. Phylogenetic Analyses

The monophyly of Scincidae was successfully recovered by both BI and ML trees, which is similar to previous studies [16,17,44,69,70]. The clade ((Sphenomorphinae + Scincinae) + Mabuyinae) of Scincidae was supported in both BI and ML trees, which is consistent with the results of Chen et al. (2021). However, this slightly contradicts the results from other studies that support the clade ((Mabuyinae + Scincinae) + Sphenomorphinae) [44,71]. Multiple studies suggest that Scincinae should be paraphyletic because *Ateuchosaurus* is nested into Lygosominae [16,18,70]. However, in the present study, although the paraphyly of Scincinae was recovered, the species of *Ateuchosaurus* was nested into Sphenomorphinae. This controversy may be related to the fact that Lygosominae was not included in our study. On the contrary, Scincinae was monophyletic and *Ateuchosaurus* was the sister to Lygosominae in a study using nuclear and mitochondrial genes by Pyron et al. [17]. When the genus *Ateuchosaurus* was not included in the phylogenetic tree, the monophyly of Scincinae was also restored [19,44,69]. In the future, samples of *Ateuchosaurus* and other skinks should be added to further explore the monophyly of Scincinae. In Mabuyinae, the phylogenetic trees constructed in this study and the results of Chen et al. (2021) based on 13 PCGs, all support that Mabuyinae was the sister of the clade (Scincinae + Sphenomorphinae). However, combined with morphological and molecular data, Mabuyinae and Sphenomorphinae showed the closest relationship [72]. Hence, the status of the Mabuyinae subfamily remains unclear due to the lack of mitochondrial genome data [44].

The species *Isopachys* clustered into a clade with the *Tropidophorus* genus and not with the *Sphenomorphus* genus, a result that is different from some previous conclusions [12,44,69,73]. Using 12S and 16S RNAs, the clades ((*Isopachys* + (*Sphenomorphus* + (*Lipinia + Scincella*))) + *Tropidophorus*) were supported in BI, ML, and maximum parsimony (MP) trees constructed by Honda et al. [73]. Although the same genes were used (12S and 16S RNA) in the phylogenetic analysis of Peninsular Malaysia skinks, Rizal et al. found that the clade of (((*Tropidophorus* + *Sphenomorphus*) + *Lipinia*) + *Isopachys*) was supported in both ML and BI trees [12]. These differences in topological structures may be related to the amount of data used to construct the phylogenetic tree. Sampling differences affecting the phylogenetic tree structure have also been found in other research [16,74,75]. The clade ((*Sphenomorphus* + *Scincella*) + *Isopachys*) was supported by using only the 13 PCGs in Chen et al. [69], whereas the tree that was constructed from 13 PCGs and 2 rRNAs (12S rRNA and 16S rRNA) showed ((*Isopachys* + *Sphenomorphus*) + *Scincella*) in Chen et al. [44]. At the same time, *Tropidophorus* was not included in the two references, and different genes used to construct the phylogenetic tree may also lead to inconsistent topological structure [16,44,69]. Thus, different samples and analytical methods will affect the results of the phylogenetic analysis. Hence, the relationship between *Isopachys*, *Sphenomorphus*, and *Tropidophorus* needs to be further verified.

### 4.2. Positive Selection Analyses

In general, positive selection acts on only a few amino acid sites over a short evolutionary period, so a signal of positive selection is usually swamped by subsequent successive negative selection at most sites in a gene sequence [76]. Similarly, after a short period of positive selection, there is usually a long purifying selection period, which neutralizes the results of the positive selection [28]. Therefore, positive selection in mitochondrial DNA is difficult to detect [28], especially in branch and clade models where ω varies only among branches and clades. On the contrary, the branch-site model and the site model are often used to distinguish the positive selection from the relaxed purification selection because they allow variations in selective pressure to occur at amino acid sites [28,76].

Consequently, analyses based on the branch-site model, suggesting a selective process that leads to the loss of limbs in *I. gyldenstolpei* was also recognizable in the ND2 gene of the mitochondrial complex I (NADH: ubiquinone oxidoreductase), which may indicate an adaptation of mitochondrial genes to the energy requirements of a limbless skink after limb loss occurs. Complex I is the first large protein complex in the respiratory chain, providing protonic power for ATP synthesis during the transfer of electrons to ubiquitin by NADH via transmembrane proton pumps. Indeed, the ND complex can drive over one-third of total energy (ATP) production in mitochondria, making it crucial in biological energy metabolism [77,78,79]. The smallest subsection of complex I is composed of the ND1-ND6 subunits that mainly form the transmembrane region core of the complex [80]. The main candidate genes for the proton pump in mitochondrial complex I are the ND2, ND4, and ND5 genes [81]. Mutations of these subunits may interfere with the efficiency of the proton pumping process [82]. In general, amino acid changes can cause inefficiencies in the electron transport chain system, which in turn affects mitochondrial energy production [82]. Certain research studies show a relationship between locomotion and energy requirements by using mitochondrial DNA. For example, it has been found that migratory fishes consume different amounts of energy compared with nonmigratory species due to differences in swimming that are reflected in the positive selections of ND1, ND3, and ND5 genes in the energy consumption of migratory fishes [24,83]. In squamates, comparisons can be made between different movement modes of limbed and limbless species. Locomotion was supported by the remaining limbs and body swing together in *Dibamus bourreti*, in which the ATP6 gene was selected compared with limbed species [33]. Furthermore, one of the major energy regulation genes in mitochondria of the limbless skink *I. gyldenstolpei* was selected (ND2 gene) during limb loss as compared with limbed skinks. This may be a relevant adaptation to the energetic requirements of *I. gyldenstolpei*. The results suggest that the difference in energy demand between limbless and limbed skinks may be related to the change of locomotion that promotes the evolution of mitochondrial DNA in different directions. However, considering the limited samples, this conclusion needs to be further verified. In future studies, we will consider adding more lizard samples to further explore whether the loss of limbs is associated with the adaptive evolution of energy-regulating genes.

## 5. Conclusions

In this study, the complete mitochondrial genome of a limbless skink, *I. gyldenstolpei*, was reported for the first time, representing the first complete mitochondrial genome of a limbless skink that has been recorded in Scincidae. At the same time, to compare with the mitochondrial genomes of limbless skinks, the complete mitochondrial genomes of two limbed skinks, *S*. *indicus* and *T*. *hainanus*, were also sequenced. Before this study, there was no complete mitochondrial genome of *T. hainanus* on NCBI, and only one complete mitochondrial genome of *S. indicus*. This study provides the complete mitochondrial genomes of *S. indicus* and *T. hainanus* to enrich the gene database of skinks. The mitochondrial genome structures of the three skinks were similar to the typical mitogenomes of reptiles. The clade of ((Sphenomorphinae + Scincinae) + Mabuyinae) in Scincidae was supported by both BI and ML analyses. The subfamily Scincinae was paraphyletic with respect to Sphenomorphinae and Mabuyinae. Our results support the conclusion that limbless *Isopachys* were more closely related to *Tropidophorus*, which as a result formed the clade (((*Isopachys* + *Tropidophorus*) + (*Sphenomorphus + Scincella*)) + *Ablepharus*). Compared with limbed skinks, one selective site was found in the mitochondrial genes of limbless *I. gyldenstolpei*. The selected site was located in the ND2 gene (position 2017, BEB = 0.907), and site 28 of the ND2 gene was located in the domain of the proton-conducting membrane transporter by the functional analysis.

## Figures and Tables

**Table 2:** Base composition of the mitochondrial genomes of the three skinks.

Region		*I. gyldenstolpei*				*S. indicus*				*T. hainanus*			
		Length (bp)	A + T (%)	AT Skew	GC Skew	Length (bp)	A + T (%)	AT Skew	GC Skew	Length (bp)	A + T (%)	AT Skew	GC Skew
mito		17,210	56	0.121	−0.358	16,944	57.9	0.11	−0.308	17,001	54.2	0.118	−0.33
PCGs	J	11,382	55.52	0.058	−0.392	11,382	57.28	0.056	−0.35	11,379	53.06	0.063	−0.363
	N			−0.651	0.602			−0.51	0.508			−0.595	0.462
rRNAs		2507	54.2	0.313	−0.207	2470	57.4	0.29	−0.147	2485	53.7	0.296	−0.191
A + T-rich region		1774	61.5	0.086	−0.46	1566	61.1	0.9	−0.359	1615	60.4	0.07	−0.404

## Data Availability

The data supporting the findings of this study are openly available from the National Center for Biotechnology Information at https://www.ncbi.nlm.nih.gov (accessed on 22 March 2022). Accession numbers are: MH020638, OM117611, and OM117612.

## Supplementary Materials

The following supporting information can be downloaded at: https://www.mdpi.com/article/10.3390/ani12162015/s1, Figure S1: The secondary structure of 22 tRNAs in the *I. gyldenstolpei* mitochondrial genome; Figure S2: The secondary structure of 22 tRNAs in the *S*. *indicus* mitochondrial genome; Figure S3: The secondary structure of 22 tRNAs in the *T*. *hainanus* mitochondrial genome; Figure S4: The protein structure of a selected limbless *I. gyldenstolpei* gene (ND2 gene) under Branch-site Model analysis and selected sites are also marked in the figure; Table S1: Specific primers used in this study to sequence the complete mitochondrial genomes of the three skink species; Table S2: The best partitioning scheme and best-fitting models were obtained by using the PartitionFinder program. The full names of all abbreviations are as follows: pos1: first codon; pos2: second codon; GTR: general time reversible; I: unchanged site proportion; G: Gamma distribution; Table S3: The localization characteristics of the mitochondrial genomes of *I. gyldenstolpei*; Table S4: The localization characteristics of the mitochondrial genomes of *S. indicus*; Table S5: The localization characteristics of the mitochondrial genomes of *T. hainanus*; Table S6: Codon numbers of the three skinks and relatively synonymous codon usage (RSCU); Table S7: The parameters and results analyzed by the branch-site model and the site model in this study; Table S8: The position and length of the nucleotides and amino acids from the 13 protein-coding genes that were used in this analysis.
