# Supplementary material for: The Complete Mitochondrial Genomes of Three Sphenomorphinae Species (Squamata: Scincidae) and the Selective Pressure Analysis on Mitochondrial Genomes of Limbless Isopachys gyldenstolpei
Source: Animals (Basel). 2022 Aug 9;12(16):2015. doi: 10.3390/ani12162015 (PMC9404441; doi:10.3390/ani12162015)
Supplement: Supplementary file 1 [file animals-12-02015-s001.zip › Table S1. Specific primers used to amplify the mitogenomes of I.gyldenstolpei, S.indicus and T.hainanus..pdf]

Table S1. Specific primers used to amplify the mitogenomes of *I. gyldenstolpei*, *S. indicus* and *T. hainanus*.

Species	Primer name	Sequence (5'-3')	Product length (bp)
I. gyldenstolpei	HDMX-J-1236	ACAGTGAAAGAGCAATGAA	3,529
	HDMX-N-4765	AGAAGAAGAGGCTGAGAAG	
	HDMX-J-5023	TAGCCCCTGAAGACCTGTA	1,265
	HDMX-N-6288	CATTTGATTATCCCTCCGT	
	HDMX-J-7183	CAAGAAGTAGAGATGGTC	2,674
	HDMX-N-9857	TAAAGTAGGTTGTGGTCA	
	HDMX-J-11430	ACACACCATCCAACTAAACC	1,441
	HDMX-N-12871	TTCCGATAAGGGCAAGAC	
	HDMX-J-14020	AAAAGGAGACGGATTAGAA	1,190
	HDMX-N-15210	AAGTAGGTGGTGGATGTGA	
	HDMX-J-16409	CGAGTTGATACTGTGATT	2,100
	HDMX-N-189	GCTAAAAGTTTGTTGAGG	
S. indicus	CXSLZ-J-764	CGTCAGGTCAAGGTGTAG	886
	CXSLZ-N-1650	CCAGGAGTTTGTGTATGC	
	CXSLZ-J-2136	GACGAGAAGACCCTGTGG	1,991
	CXSLZ-N-4127	GCTGGTTGGTTTGTTAGTTG	
	CXSLZ-J-5602	TTCTATTAGCCTCCTCAG	1,519
	CXSLZ-N-7121	ATTGTCCAGATTATTTCG	
	CXSLZ-J-10534	GCTATCCGCTGGAGTCTA	1,630
	CXSLZ-N-12164	GAGGTGTTTGCGTCTGAG	
	CXSLZ-J-13449	CTCATCAAAGCCTATCTAA	1,104
	CXSLZ-N-14553	AACTCTGTTCCAATGTAAG	
	CXSLZ-J-16234	GCCTGTCAGTTCCGTTCC	2,500
	CXSLZ-N-335	CATTGTTTTTGGTCTTTCGTAT	
T. hainanus	HNLXX-J-570	ATGCCCACCACAACATTATC	851
	HNLXX-N-1421	GGCTTTTCACCTCTACTACG	
	HNLXX-J-1971	GCAACACAAGTATCAGGGGT	2,336
	HNLXX-N-4307	GTGGTGGGTTAGGTAGAGGA	
	HNLXX-J-4968	CAAAGCCGCAAACAAGAGT	1,779
	HNLXX-N-6747	CAGCCGTGTAGTCATTCAAG	
	HNLXX-J-7601	CAATCGTTGTAGAGACTGT	3,573
	HNLXX-N-11174	CAAGATGTTATTAGGGGTA	
	HNLXX-J-11192	CTACCCCTAATAACATCTTG	1,984
	HNLXX-N-13176	TGTGACGAGTAATGCTGA	
	HNLXX-J-16200	TGGTCGCCTGTCAGTTCC	2,000
	HNLXX-N-353	TTTACGCCGTGGGTCATT	
