# Supplementary material for: The Complete Mitochondrial Genomes of Three Sphenomorphinae Species (Squamata: Scincidae) and the Selective Pressure Analysis on Mitochondrial Genomes of Limbless Isopachys gyldenstolpei
Source: Animals (Basel). 2022 Aug 9;12(16):2015. doi: 10.3390/ani12162015 (PMC9404441; doi:10.3390/ani12162015)
Supplement: Supplementary file 1 [file animals-12-02015-s001.zip › Table S2. The best partitioning scheme and best-fitting models are obtained by using PartitionFinder program.pdf]

Table S2. The best partitioning scheme and best-fitting models are obtained by using PartitionFinder program. The full names of all abbreviations are as follows: pos1: first codon; pos2: second codon; GTR: general time reversible; I: unchanged site proportion; G: Gamma distribution.

Nucleotide Sequence Alignments		
**Subset**	**Subset Partitions**	**Best Model**
Partition 1	ND3_pos1, ND1_pos1, ND4_pos1, ATP6_pos1, ND4l_pos1, ND2_pos1, ND5_pos1, ATP8_pos1, ATP8_pos2	GTR+I+G
Partition 2	ND3_pos2, ATP6_pos2, ND4l_pos2, ND2_pos2, ND4_pos2, ND5_pos2	TVM+I+G
Partition 3	COI_pos1	TRNEF+I+G
Partition 4	COI_pos2	F81+I
Partition 5	CYTB_pos1, COIII_pos1, COII_pos1	TVMEF+I+G
Partition 6	COII_pos2, COIII_pos2, CYTB_pos2, ND1_pos2	TVM+I+G
Partition 7	ND6_pos2, ND6_pos1	HKY+G
