# Supplementary material for: The Complete Mitochondrial Genomes of Three Sphenomorphinae Species (Squamata: Scincidae) and the Selective Pressure Analysis on Mitochondrial Genomes of Limbless Isopachys gyldenstolpei
Source: Animals (Basel). 2022 Aug 9;12(16):2015. doi: 10.3390/ani12162015 (PMC9404441; doi:10.3390/ani12162015)
Supplement: Supplementary file 1 [file animals-12-02015-s001.zip › Table S3. Location of features in the mitochondrial genome of I. gyldenstolpei.pdf]

Table S3. Location of features in the mitochondrial genome of *I. gyldenstolpei*.

Gene/region	Start Position	Stop position	Intergenic nucleotides	Length (bp)	Start codon	Stop codon	Strand
$tRNA^{Phe}$	1	73		73			H
12S rRNA	74	1015		942			H
$tRNA^{Val}$	1016	1085		70			H
16S rRNA	1086	2613		1528			H
$tRNA^{Leu(UUR)}$	2614	2688		75			H
ND1	2689	3651	4	963	ATG	TAA	H
$tRNA^{Ile}$	3656	3724	2	69			H
$tRNA^{Gln}$	3727	3797	-1	71			L
$tRNA^{Met}$	3797	3865		69			H
ND2	3866	4900	-2	1035	ATG	TAG	H
$tRNA^{Trp}$	4899	4967		69			H
$tRNA^{Ala}$	4968	5036		69			L
$tRNA^{Asn}$	5037	5109	13	73			L
$tRNA^{Cys}$	5123	5186		64			L
$tRNA^{Tyr}$	5187	5252	1	66			L
COI	5254	6801	-5	1548	GTG	AGA	H
$tRNA^{Ser(UCN)}$	6797	6867	3	71			L
$tRNA^{Asp}$	6871	6938		68			H
COII	6939	7626		688	ATG	T	H
$tRNA^{Lys}$	7627	7691	2	65			H
ATP8	7694	7858	-10	165	ATG	TAA	H
ATP6	7849	8532	-1	684	ATG	TAA	H
COIII	8532	9315		784	ATG	T	H
$tRNA^{Gly}$	9316	9383		68			H
ND3	9384	9731	-2	348	ATG	TAA	H
$tRNA^{Arg}$	9730	9798		69			H
ND4L	9799	10095	-7	297	ATG	TAA	H
ND4	10089	11469		1381	ATG	T	H
$tRNA^{His}$	11470	11537		68			H
$tRNA^{Ser(AGY)}$	11538	11603	-1	66			H
$tRNA^{Leu(CUN)}$	11603	11674	1	72			H
ND5	11676	13502	-5	1827	ATG	TAA	H
ND6	13498	14019		522	ATG	AGG	L
$tRNA^{Glu}$	14020	14087	2	68			L
Cyt b	14090	15232	6	1143	ATG	TAA	H
$tRNA^{Thr}$	15239	15309		71			H
$tRNA^{Pro}$	15310	15378		69			L
D-loop	15379	16944		1566			H
