# Supplementary material for: The Complete Mitochondrial Genomes of Three Sphenomorphinae Species (Squamata: Scincidae) and the Selective Pressure Analysis on Mitochondrial Genomes of Limbless Isopachys gyldenstolpei
Source: Animals (Basel). 2022 Aug 9;12(16):2015. doi: 10.3390/ani12162015 (PMC9404441; doi:10.3390/ani12162015)
Supplement: Supplementary file 1 [file animals-12-02015-s001.zip › Table S5. Location of features in the mitochondrial genome of T. hainanus.pdf]

Table S5. Location of features in the mitochondrial genome of *T. hainanus*.

Gene/region	Start Position	Stop position	Intergenic nucleotides	Length (bp)	Start codon	Stop codon	Strand
tRNA^{Phe}	1	73		73			H
12S rRNA	74	1031		958			H
tRNA^{Val}	1032	1101		70			H
16S rRNA	1102	2628		1527			H
tRNA$^{Leu(UUR)}$	2629	2703		75			H
ND1	2704	3666	4	963	GTG	TAA	H
tRNA^{Ile}	3671	3742	3	72			H
tRNA^{Gln}	3746	3816	-1	71			L
tRNA^{Met}	3816	3884		69			H
ND2	3885	4919	-2	1035	ATG	TAG	H
tRNA^{Trp}	4918	4988		71			H
tRNA^{Ala}	4989	5057		69			L
tRNA^{Asn}	5058	5130	13	73			L
tRNA^{Cys}	5144	5207		64			L
tRNA^{Tyr}	5208	5274	1	67			L
COI	5276	6823	-5	1548	GTG	AGA	H
tRNA$^{Ser(UCN)}$	6819	6889	3	71			L
tRNA^{Asp}	6893	6960	1	68			H
COII	6962	7649		688	ATG	T	H
tRNA^{Lys}	7650	7714	1	65			H
ATP8	7716	7883	-10	168	ATG	TAA	H
ATP6	7874	8557	-1	684	ATG	TAA	H
COIII	8557	9340		784	ATG	T	H
tRNA^{Gly}	9341	9409		69			H
ND3	9410	9757	-2	348	ATG	TAG	H
tRNA^{Arg}	9756	9824		69			H
ND4L	9825	10121	-7	297	ATG	TAA	H
ND4	10115	11495		1381	ATG	T	H
tRNA^{His}	11496	11564		69			H
tRNA$^{Ser(AGY)}$	11565	11630	-1	66			H
tRNA$^{Leu(CUN)}$	11630	11700	1	72			H
ND5	11702	13525	-5	1824	ATG	TAA	H
ND6	13521	14042		522	ATG	AGG	L
tRNA^{Glu}	14043	14110	-2	68			L
Cyt b	14109	15251	-1	1143	ATG	TAA	H
tRNA^{Thr}	15251	15319		69			H
tRNA^{Pro}	15320	15386		67			L
D-loop	15387	17001		1615			H
