# Supplementary material for: The Complete Mitochondrial Genomes of Three Sphenomorphinae Species (Squamata: Scincidae) and the Selective Pressure Analysis on Mitochondrial Genomes of Limbless Isopachys gyldenstolpei
Source: Animals (Basel). 2022 Aug 9;12(16):2015. doi: 10.3390/ani12162015 (PMC9404441; doi:10.3390/ani12162015)
Supplement: Supplementary file 1 [file animals-12-02015-s001.zip › Table S6. Codon numbers and relative synonymous codon usage (RSCU) in mitochondrial protein coding genes of I.gyldenstolpei (IG), S.indicus (SI) and T.hainanus (TH)..pdf]

Table S6. Codon numbers and relative synonymous codon usage (RSCU) in mitochondrial protein coding genes of *I.gyldenstolpei* (IG), *S.indicus* (SI) and *T.hainanus* (TH).

**Codon**	**Count**			**RSCU**			**Codon**	**Count**			**RSCU**		
	IG	SI	TH	IG	SI	TH		IG	SI	TH	IG	SI	TH
**UUU(F)**	118	122	94	1.09	1.1	0.9	**GCA(A)**	93	109	81	1.31	1.49	1.04
**UUC(F)**	99	100	114	0.91	0.9	1.1	**GCG(A)**	8	12	23	0.11	0.16	0.3
**UUA(L)**	91	142	93	0.84	1.34	0.85	**UAU(Y)**	46	51	38	0.88	0.89	0.7
**UUG(L)**	42	27	32	0.39	0.25	0.29	**UAC(Y)**	59	63	71	1.12	1.11	1.3
**CUU(L)**	112	121	87	1.03	1.14	0.8	**CAU(H)**	23	26	16	0.39	0.48	0.29
**CUC(L)**	127	67	125	1.17	0.63	1.15	**CAC(H)**	95	83	94	1.61	1.52	1.71
**CUA(L)**	213	231	249	1.97	2.17	2.28	**CAA(Q)**	93	84	85	1.88	1.77	1.68
**CUG(L)**	65	50	69	0.6	0.47	0.63	**CAG(Q)**	6	11	16	0.12	0.23	0.32
**AUU(I)**	133	140	87	0.98	0.98	0.65	**AAU(N)**	29	41	29	0.42	0.59	0.45
**AUC(I)**	139	145	180	1.02	1.02	1.35	**AAC(N)**	110	97	101	1.58	1.41	1.55
**AUA(M)**	134	156	140	1.43	1.49	1.36	**AAA(K)**	67	78	67	1.7	1.79	1.65
**AUG(M)**	53	53	66	0.57	0.51	0.64	**AAG(K)**	12	9	14	0.3	0.21	0.35
**GUU(V)**	58	51	38	1.22	1.1	0.85	**GAU(D)**	15	18	20	0.46	0.53	0.57
**GUC(V)**	39	48	54	0.82	1.03	1.21	**GAC(D)**	50	50	50	1.54	1.47	1.43
**GUA(V)**	71	63	63	1.49	1.35	1.41	**GAA(E)**	64	59	54	1.51	1.37	1.27
**GUG(V)**	22	24	24	0.46	0.52	0.54	**GAG(E)**	21	27	31	0.49	0.63	0.73
**UCU(S)**	50	38	39	1.18	0.9	0.9	**UGU(C)**	12	8	8	0.92	0.55	0.5
**UCC(S)**	61	51	74	1.44	1.21	1.71	**UGC(C)**	14	21	24	1.08	1.45	1.5
**UCA(S)**	83	103	88	1.95	2.45	2.04	**UGA(W)**	82	86	80	1.55	1.56	1.48
**UCG(S)**	14	12	9	0.33	0.29	0.21	**UGG(W)**	24	24	28	0.45	0.44	0.52
**CCU(P)**	40	28	31	0.75	0.54	0.56	**CGU(R)**	3	11	5	0.17	0.63	0.28
**CCC(P)**	61	45	82	1.15	0.87	1.49	**CGC(R)**	17	10	17	0.99	0.57	0.96
**CCA(P)**	104	119	83	1.96	2.29	1.51	**CGA(R)**	45	41	42	2.61	2.34	2.37
**CCG(P)**	7	16	24	0.13	0.31	0.44	**CGG(R)**	4	8	7	0.23	0.46	0.39
**ACU(T)**	41	40	38	0.41	0.44	0.43	**AGU(S)**	12	7	9	0.28	0.17	0.21
**ACC(T)**	150	127	137	1.51	1.41	1.57	**AGC(S)**	35	41	40	0.82	0.98	0.93
**ACA(T)**	198	180	157	1.99	1.99	1.79	**GGU(G)**	31	30	15	0.54	0.53	0.26
**ACG(T)**	8	14	18	0.08	0.16	0.21	**GGC(G)**	86	77	85	1.5	1.37	1.47
**GCU(A)**	31	32	45	0.44	0.44	0.58	**GGA(G)**	71	66	57	1.23	1.17	0.99
**GCC(A)**	152	139	162	2.14	1.9	2.08	**GGG(G)**	42	52	74	0.73	0.92	1.28
