# Supplementary material for: The Complete Mitochondrial Genomes of Three Sphenomorphinae Species (Squamata: Scincidae) and the Selective Pressure Analysis on Mitochondrial Genomes of Limbless Isopachys gyldenstolpei
Source: Animals (Basel). 2022 Aug 9;12(16):2015. doi: 10.3390/ani12162015 (PMC9404441; doi:10.3390/ani12162015)
Supplement: Supplementary file 1 [file animals-12-02015-s001.zip › Table S7. The parameters and results analyzed by the Branch-site model and the Site Model in this study.pdf]

Table S7. The parameters and results analyzed by the Branch-site model and the Site Model in this study (*P* < 0.05 indicates significant difference).

Analysis Model	Tree	Foreground	Model	LnL	Estimates of Parameters					Model Compared	***LRT P*-value**	Positive sites
**Branch-site Model**		*I. gyldenstolpei*	Model A	-94573.14	Site class	0	1	2a	2b	Model A vs. Model A null	0.02189855	2017 L 0.907
					f	0.892	0.1	0.005	0.0006			
					ω0	0.0417	1	0.042	1			
					ω1	0.0417	1	51.02	51.021			
			Model A null	-94575.77	1							/
			**Model**	**LnL**	**Estimates of Parameters**					**Model Compared**	***LRT P*-value**	**Positive sites**
**Branch Model**	BI		M3	-93035.23	p:	0.7265	0.27	0		M0 vs. M3	0	/
					ω:	0.0088	0.24	51.27812				
			M0	-96045.71	ω0:	0.0676						
			M2a	-94578.86	p:	0.8968	0.1	0		M1a vs. M2a	0.99970404	/
					ω:	0.042	1	30.00557				
			M1a	-94578.86	p:	0.8968	0.1					
					ω:	0.042	1					
			M8	-92940.96	p0=0.98041 (p1= 0.01959)	p=0.20947 ω= 1.00000	q=1.97464			M7 vs.M8	0	2416 Q 0.931;3742 G 0.960*
			M7	-93024.32	p=	0.2161	q=	1.71599				/
