# Supplementary material for: The Complete Mitochondrial Genomes of Three Sphenomorphinae Species (Squamata: Scincidae) and the Selective Pressure Analysis on Mitochondrial Genomes of Limbless Isopachys gyldenstolpei
Source: Animals (Basel). 2022 Aug 9;12(16):2015. doi: 10.3390/ani12162015 (PMC9404441; doi:10.3390/ani12162015)
Supplement: Supplementary file 1 [file animals-12-02015-s001.zip › Table S8. The order and length of nucleotide and amino acid from 13 protein-coding genes were used in the analysis..pdf]

Table S8. The order and length of nucleotide and amino acid from 13 protein-coding genes were used in the analysis.

Gene	Nucleotide position	Length of nucleotide sequence	Amino acid position	Length of amino acid sequence
ATP6	1-681	681 bp	1-227	227
ATP8	682-846	165 bp	228-282	55
COX1	847-2,391	1,545 bp	283-797	515
COX2	2,392-3,078	687 bp	798-1,026	229
COX3	3,079-3,861	783 bp	1,027-1,287	261
CYTB	3,862-5,004	1143 bp	1,288-1,668	381
ND1	5,005-5,967	963 bp	1,669-1,989	321
ND2	5,968-6,999	1,032 bp	1,990-2,333	344
ND3	7,000-7,344	345 bp	2,334-2,449	115
ND4L	7,345-7,638	294 bp	2,449-2,531	83
ND4	7,639-9,018	1,380 bp	2,532-2,991	460
ND5	9,019-10,842	1,824 bp	2,992-3,599	608
ND6	10,843-11,373	531 bp	3,600-3,776	177
